# How disability severity is associated with changes in physical activity and inactivity from adolescence to young adulthood

**DOI:** 10.1186/s13690-023-01043-0

**Published:** 2023-02-21

**Authors:** Jihoon Jung, Seungyeon Park, Chung Gun Lee

**Affiliations:** 1 Department of City and Regional Planning, University of North Carolina at Chapel Hill, Chapel Hill, NC USA; 2 Department of Health, Physical Education & Exercise Science, Norfolk State University, Norfolk, VA USA; 3 Department of Physical Education, Seoul National University, Seoul, South Korea

**Keywords:** Physical activity, Physical inactivity, Disability, Adolescence, Young adulthood, Transition

## Abstract

**Background:**

Disabilities may play a different role in determining people’s physical activity (PA) and physical inactivity (PI) levels when they go through multiple lifetime transitions (e.g., graduation, marriage) between adolescence and young adulthood. This study investigates how disability severity is associated with changes in PA and PI engagement levels, focusing on adolescence and young adulthood, when the patterns of PA and PI are usually formed.

**Methods:**

The study employed data from Waves 1 (adolescence) and 4 (young adulthood) of the National Longitudinal Study of Adolescent Health, which covers a total of 15,701 subjects. We first categorized subjects into 4 disability groups: no, minimal, mild, or moderate/severe disability and/or limitation. We then calculated the differences in PA and PI engagement levels between Waves 1 and 4 at the individual level to measure how much the PA and PI levels of individuals changed between adolescence and young adulthood. Finally, we used two separate multinomial logistic regression models for PA and PI to investigate the relationships between disability severity and the changes in PA and PI engagement levels between the two periods after controlling for multiple demographic (age, race, sex) and socioeconomic (household income level, education level) variables.

**Results:**

We showed that individuals with minimal disabilities were more likely to decrease their PA levels during transitions from adolescence to young adulthood than those without disabilities.

Our findings also revealed that individuals with moderate to severe disabilities tended to have higher PI levels than individuals without disabilities when they were young adults. Furthermore, we found that people above the poverty level were more likely to increase their PA levels to a certain degree compared to people in the group below or near the poverty level.

**Conclusions:**

Our study partially indicates that individuals with disabilities are more vulnerable to unhealthy lifestyles due to a lack of PA engagement and increased PI time compared to people without disabilities. We recommend that health agencies at the state and federal levels allocate more resources for individuals with disabilities to mitigate health disparities between those with and without disabilities.

**Supplementary Information:**

The online version contains supplementary material available at 10.1186/s13690-023-01043-0.

## Background

Increases in physical activity (PA) are highly associated with increased energy expenditure, better body composition (i.e., less fat), an improved immune system, improved cardiovascular functions, and other physiological effects (e.g., appetite control) [1, 2]. On the other hand, a lack of PA or increases in physical inactivity (PI) could result in elevated risks of a wide range of adverse health outcomes, including obesity, diabetes, cardiovascular disease, bone health, and mental health [3–7]. One recent study showed that 8.3% of deaths could have been prevented with an adequate level of PA [8]. Another study also supports that physically inactive people have a 20–30% higher risk of more than 25 chronic medical issues and premature death than physically active individuals [9]. Despite the importance of PA, over 31% of the world’s population and over 80% of the world’s adolescent population do not satisfy the minimum PA recommendations [10].

The individual levels of PA and PI are determined by a wide range of demographic factors. Age is one of the major factors associated with PA and PI levels. Previous studies have shown that individuals over 65 years of age are less likely to engage in PA than individuals in other age groups, probably because of their relatively reduced physiological functions, such as increased fatness, weaker muscles, lower bone density, and decreased cardiorespiratory and metabolic functions [11–13]. Those over 65 years of age are also more likely to experience fall-related accidents, which can cause injuries and limit PA engagement [14]. In addition, a recent meta-analysis indicated that the PA of both males and females decreased during the transition from adolescence into young adulthood [15]. However, Gordon-Larsen et al. (2006) also argued that PA and sedentary behavior patterns during adolescence are likely to continue into adulthood [16].

Sex is another important factor influencing the level of PA participation. Multiple studies have indicated that males tend to be more active in regular PA and leisure activities than females [17–20]. There are several explanations for this PA gap between the two sexes. First, psychological factors differently affect PA participation. For instance, young adult males tend to have intrinsic motivations for PA and exercise such as enjoyment and a challenging spirit, while young adult females tend to have more extrinsic motivations such as weight loss and improving one’s body image [21, 22]. The lack of motivation, lack of confidence, and low self-efficacy for PA in females during childhood further influence their future level of PI during adulthood [23]. Other factors, such as relatively fewer PA options for girls, could result in demotivation to PA participation when they transition from a young age group into adulthood [24]. Sex differences in PA exist regardless of race/ethnicity, socioeconomic status (SES), and age [17, 25, 26].

Previous studies also found that individuals of several races and ethnicities were less physically active than those of other races, such as Asian individuals, Hispanic non-White female young adults, Hispanic immigrant children, and African American female young adults [17, 27–31]. This difference may be associated with SES rather than genetic or physiological differences. Bantham et al. (2021) showed that Blacks and Latinos had more economic difficulties in accessing health care, and their poor health conditions also impacted their PI [32]. Different societal and cultural expectations could lead to additional differences in exercise motives by race/ethnicity [33]. For example, McArthur and Raedeke (2009) found that female and White college students put more emphasis on appearance-related motives than male and Black students [34]. This could be partially due to different cultural expectations. Walcott-McQuigg et al. (1995) reported that Black female college students expressed beliefs that Black males prefer a more rounded appearance [35].

In addition, socioeconomic factors play an important role in deciding the rate of PA participation. Previous studies enumerated diverse variables that could impact the PA engagement of individuals with low-SES. Giles-Corti et al. (2002) found that individuals residing in low-SES communities were approximately 36% less active due to their environmental constraints [36]. Low-income could have a negative impact on PA levels [37]. A lack of facilities, a lack of economic affordability, or safety concerns in neighborhoods adds additional barriers to PA engagement [16, 38]. Parental education level is also related to the PA participation levels of children. For instance, adolescents or children with parents with a higher education level had significantly higher PA participation than those with parents with lower education levels [39–41].

Physical disability and/or limitation status may be one of the most important factors highly associated with PA and PI patterns. Previous literature has continuously indicated that people with disabilities tend to have lower PA and higher PI rates than the general population [42–45]. Individuals with disabilities are more likely to face environmental and individual barriers to PA participation than the general population. Environmental factors including the built environment, SES, psychological status, and the perceptions of other people influence the PA levels of those with disabilities [46]. Individual factors such as self-efficacy, attitude, motivation, age, and the severity of disabilities and physical limitations impact the types and intensity of PA as well [13, 47, 48]. For example, as age increases, young adults with disabilities make more independent and subjective PA decisions [48].

Most of the previous studies have only focused on the differences in PA and PI levels between people with and without disabilities, which does not take the impact of age into account. However, people with and without disabilities may have different experiences while going through multiple transitions, such as graduation, marriage, living apart from one’s parents, and starting work. These different experiences would introduce disparities in PA levels over the life course between individuals with and without disabilities [49]. In addition, to the best of our knowledge, no studies have looked at the impact of disabilities on PI changes over the life course, even though disability/limitation status could have different impacts on both PA and PI levels in individuals in different age groups [50]. In this respect, our study attempted to understand how the level of disability is associated with changes in PA and PI engagement levels over the life course. For this, we separately regressed the changes in PA and PI levels between adolescents and young adults with four different severity levels of disability/limitation status (i.e., no, minimal, mild, and moderate/severe disability and/or physical limitation) after controlling for multiple demographic (age, race, sex) and socioeconomic (household income level, education level) variables. Here, we were especially interested in two periods, adolescence and young adulthood, when the patterns of PA and PI are usually formed. Young adulthood, when people reach physical maturity, is regarded as an important transition period that has many lifetime and transition events, such as leaving education, living apart from one’s parents, starting work, and getting married. These transition events usually influence PA and PI engagement rates in this period [51–53]. We believe our comprehensive models including multiple demographic and socioeconomic variables could give more accurate and robust results to understand the impact of physical disability and/or limitation status on changes in PA and PI levels over the life course.

### Data and methodology

Overall, we used three types of datasets obtained from the National Longitudinal Study of Adolescent Health (https://addhealth.cpc.unc.edu): 1) physical disability and/or limitation; 2) demographic and socioeconomic variables; and 3) PA and PI. With these datasets, we first divided all subjects into four groups by disability level: no, minimal, mild, and moderate/severe disability and/or limitation. We then investigated how the severity of disability impacted the changes in lifetime PA and PI levels with multinomial logistic regressions. To control for the impacts of other factors, we included three demographic (age, race, sex) and two socioeconomic (household income level, education level) variables in the models. More details are as follows.

### Wave data

The aim of the National Longitudinal Study of Adolescent Health, also called Add Health, is to investigate overall health-related variables following a sequence of developmental shifts: adolescence, young adulthood, and later adulthood. The first Add Health survey (Wave 1) was completed between 1994 and 1995. There were 20,745 adolescent respondents in grades 7 through 12 included in Wave 1. Follow-up Add Health surveys, called Waves 2–5, have been conducted over the last 20 years. The most recent survey (Wave 5) was conducted during 2016–2018. Although recent Waves (Waves 4 and 5) include more variables, including educational transitions, SES, and sleep health, mostly similar questions on health, education, nutrition, emotion, family, and neighborhood structure have been asked across the Waves.

In the present study, we selected Waves 1 (ages: 12–18 years) and 4 (ages: 24–32 years) to observe changes in PA and PI levels between adolescence and young adulthood, when the patterns of PA and PI are usually formed. Since only respondents who engaged in Wave 1 were eligible for Wave 4, we were able to track the same individuals with an approximately 15-year time gap. The total number of subjects included in the study was 15,701. From Waves 1 and 4, we extracted three datasets. Physical disability and/or limitation data were obtained from only Wave 1 since disability is a permanent condition and rarely changes over time. In addition, Wave 1 included a wider range of questions on disabilities and physical limitations, including limb difficulties, the use of equipment, personal care assistance, blindness, and deafness, than Wave 4. For demographic and socioeconomic variables, we only used Wave 4 because we wanted to investigate the impact of these variables on current PA and PI levels. The last datasets, the PA and PI datasets, were downloaded from both Waves 1 and 4 to observe the changes in PA and PI levels between the two periods. More details on data preparation for disability and PA/PI levels can be found below.

## Methodology

### Multinomial logistic regression

We used multinomial logistic regression to investigate how physical disabilities and/or limitations influence changes in PA and PI engagement levels between adolescents and younger adults using the same subjects after controlling for multiple covariates (i.e., age, race, sex, household income level, education level). Multinomial logistic regression is a simple extension of binomial logistic regression, which allows for more than two dependent variable categories (e.g., excellent, very good, good, fair, poor). This regression model uses the maximum likelihood estimate to predict the probability that an observation or respondent falls into one of the multiple dependent variable categories (either nominal or unordered) based on one or more independent variables (either continuous or categorical). Unlike other multivariate analysis models, multinomial logistic regression does not assume normality, linearity between the dependent and independent variables, homoscedasticity, or normally distributed error terms (residuals) [54].

The results of multinomial regressions show the impact of independent variables on the probability of falling into one of the multiple dependent variable categories in comparison to the reference category [55]. This method commonly uses relative risk ratios (RRRs) to describe model results. An RRR shows the risk of the outcome falling in the comparison group compared to the risk of the outcome falling in the reference group. An RRR > 1 suggests that the risk of the outcome occurring in the comparison group is higher (more likely) than the risk of the outcome occurring in the reference group. On the other hand, an RRR < 1 indicates that the risk of the outcome occurring in the comparison group is lower (less likely) than the risk of the outcome occurring in the reference group. In other words, for an RRR > 1, a comparison outcome is more likely, while for an RRR < 1, a reference outcome is more likely. For example, an RRR of 2 means that the comparison outcome is 2 times more likely than the reference outcome, while an RRR of 0.5 means that the comparison outcome is 0.5 times less likely than the reference outcome.

In this study, we initially checked multicollinearity between independent variables to avoid incorrect or inflated coefficients (no violation was found). Then, we separately performed two multinomial logistic regressions with the change in PA and PI engagement levels between Waves 1 and 4 as the dependent variable. The independent variables in the models were disability severity (no, minimal, mild, and moderate/severe disability and/or limitation), demographic (age, race, sex) variables, and socioeconomic (household income level, education level) variables. This study was classified as exempt by the Norfolk State University Institutional Review Board.

### Physical disability score

**Fig. 1:**
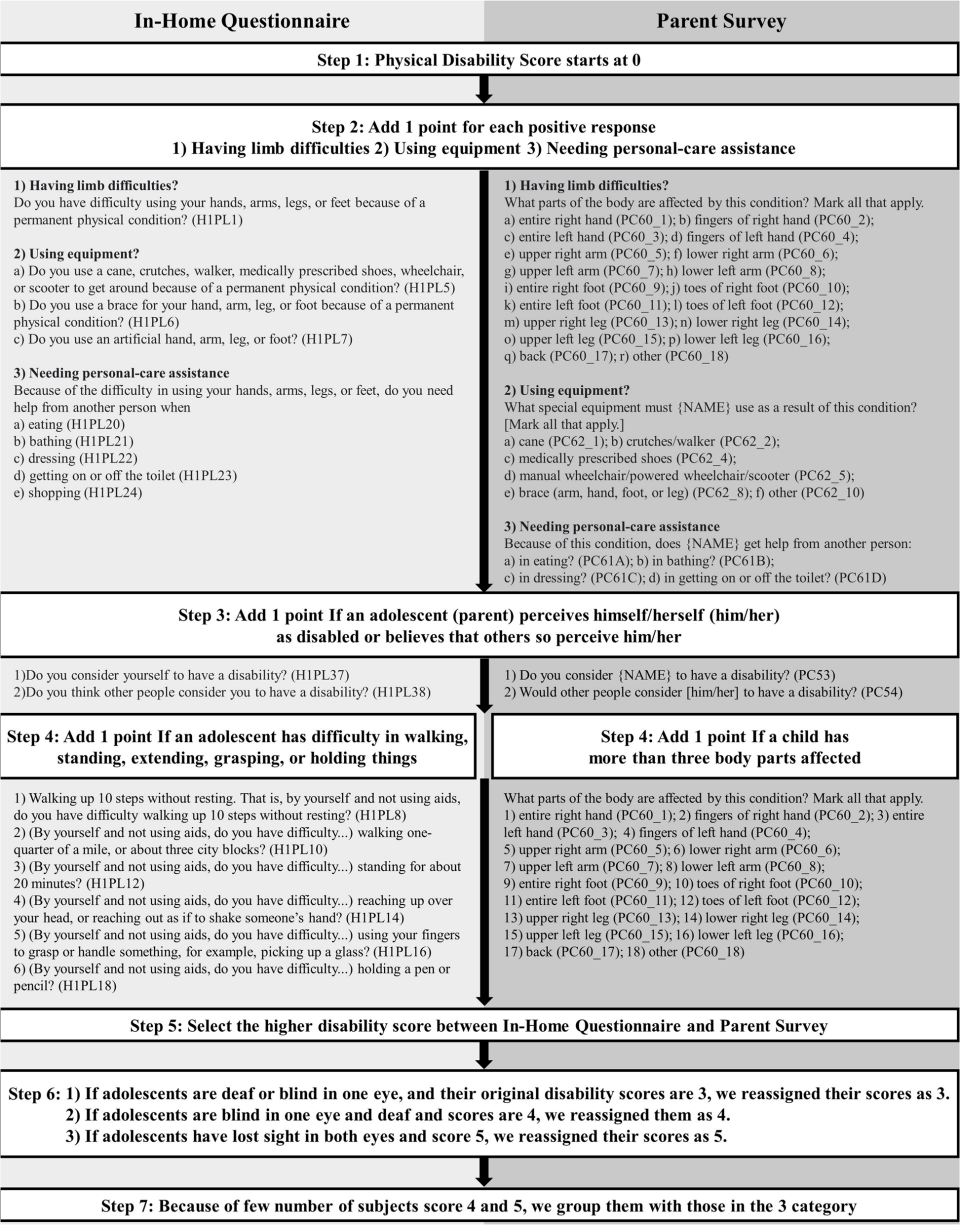
Diagram of how we assigned physical disability scores

Wave 1 included the In-Home Questionnaire and Parent Survey. Both surveys contain multiple questions on physical disabilities and/or limitations, providing information on (a) the specifics of an individual’s disabilities, (b) the extent of their functional limitations and (c) whether personal care assistance is needed. We followed previous studies to assign physical disability scores to each individual [56, 57]. Figure 1 shows how we categorized physical disabilities and/or limitations. The process of assigning a physical disability score has 7 steps. We separately calculated the physical disability scores with the In-Home Questionnaire and Parent Survey until Step 4, and then we selected the higher score between the two surveys at Step 5. With the selected scores, we conducted Steps 6 and 7. More details on the classification process can be found in Fig. 1.

### Measuring physical activity and inactivity

The PA level was determined based on total bouts of moderate to vigorous PA per week. A number of previous studies used this method to categorize moderate to vigorous PA using a standard 1-week recall questionnaire [58–61]. As our main foci were moderate to vigorous PA, we only included questions that were matched to moderate to vigorous PA levels (e.g., 5 to 8 metabolic equivalents; exercise and sports), excluding any questions associated with mild-intensity or low-intensity PA (2 to 3 metabolic equivalents; hobbies and cleaning). In Wave 1 Sect. 2 (4, 5, and 6), respondents were asked how many times per week they spent performing specific physical activities (e.g., inline skating, skateboarding, bicycling, football, basketball, baseball, soccer, softball, swimming, jogging, walking, martial arts, jump roping, dancing, and gymnastics). Likewise, in Wave 4 Sect. 25 (2–8), similar questions were asked. The only difference was that the same questions from Wave 1 were divided into more specific questions according to the types of activities in Wave 4. The PI level was also decided based on the total amount of time for several activities which was drawn from a 1-week recall questionnaire. PI questions included the total amount of time spent watching television, watching movies, playing computer games, and listening to music on a weekly basis. We utilized the total amount of time spent on these physically inactive and sedentary behaviors. This is one of the most commonly used ways to ask PI-related questions in surveys from previous studies [60]. Here, please note that the increase in PA does not necessarily mean the decrease in PI as they are independent. Relatedly, the sum of PA and PI is not fixed (not zero-sum) because the questions used for PA and PI in the questionnaire do not cover all daily activities. For example, the questionnaire does not cover time for studying, working, sleeping, eating, etc. Our data actually shows very weak Pearson correlation coefficients between the sum of PA and PI (Wave 1: 0.02; Wave 4: -0.04). Thivel et al. (2018) also support that PA and PI are not the opposite of each other [62]. More information on the survey questions used to collect the level of PA and PI can be found in Supplementary Table 1.

To calculate the relative changes in PA and PI between adolescence and young adulthood, we first extracted PA and PI data from both Waves 1 (adolescence) and 4 (young adulthood). We then separately summed up the total frequency of PA and total time of PI for Waves 1 and 4. Next, we normalized the sum of PA and PI with min–max normalization for each Wave data (Eq. 1).

1 $${x}_{normalized} = \frac{(x - {x}_{min})}{({x}_{max} - {x}_{min})}$$

Where x_normalized_ is the normalized value; x is the initial value; x_max_ and x_min_ stand for the maximum and minimum value. This process is necessary because Waves 1 and 4 data did not use the same questionnaires for PA and PI. In addition, the level of PA and PI would be different between adolescence and young adulthood. For example, our survey data shows different mean hours in PI between Wave 1 (23.0 h) and Wave 4 (16.2 h) even though no differences were found in PA (Wave 1:3.6 times, Wave 4: 3.6 times). A direct comparison without a normalization process would lead to the wrong results that all people decrease PI levels between adolescence and young adulthood. Based on the normalized total frequency of PA and time of PI, we were able to calculate the changes in PA and PI between two Waves. Finally, we reclassified the differences into five relative categories for easy interpretation with standard deviations (SDs): 1) < -2SDs, 2) -2SDs ~ -1SD, 3) -1SD ~  + 1SD, 4) + 1SD ~  + 2SDs, and 5) >  + 2SDs. Here, the category “ < -2SDs” represents the group with the highest decreases in PA and PI levels between the two survey periods, and the category “ >  + 2SDs” represents the group with the highest increases in PA and PI levels. On the other hand, the category “-1SD ~  + 1SD” represents the group with no changes in PA and PI levels between the two periods. For intuitive interpretation, we renamed these five categories as follows: 1) more decreased (< -2SDs), 2) decreased (-2SDs ~ -1SD), 3) no change (-1SD ~  + 1SD), 4) increased (+ 1SD ~  + 2SDs), and 5) more increased (> + 2SDs).

## Results

In this study, we aimed to understand how the severity of disability (i.e., no, minimal, mild, and moderate/severe disability and/or limitation) is associated with changes in PA and PI levels from adolescence through young adulthood after controlling for multiple demographic and socioeconomic variables. In the results, we first report descriptive summaries of the subjects’ physical disabilities and/or limitations, demographic and socioeconomic variables, and PA and PI engagement levels. We then show the associations between changes in PA/PI levels and multiple demographic and socioeconomic variables. Finally, we exhibit how physical disability and/or limitation status influences changes in PA and PI levels between adolescence and young adulthood.

### Descriptive summary

Table 1 summarizes the physical disabilities and limitations of adolescent respondents in Wave 1. We categorized the study participants into four levels by disability severity: no, minimal, mild, and moderate/severe disability and/or limitation. Among 15,701 subjects, there were 14,783 respondents without disabilities (94%), 517 adolescents with a minimal level of disability (3%), 185 adolescents with a mild level of disability (1%), and 216 adolescents with a moderate/severe level of disability (1%). Overall, individuals with disabilities (i.e., In-Home Questionnaire) themselves were more likely than their parents to report that they had limb difficulties, used equipment for their conditions, and needed aids for daily activities (i.e., Parent Survey). However, individuals with disabilities were less likely than their parents to answer that they or others considered themselves to have a disability. This gap decreased as disability severity increased. For example, while the same number (*n* = 83) of individuals with more moderate/severe disability and their parents reported that they considered themselves or their children as disabled, only 5 out of the 517 people with minimal disability and 23 out of the 517 parents answered that they or their child had a disability.

**Table 1:** Descriptive summary of physical disabilities and/or limitation in Wave 1

	No	Minimal	Mild	Moderate/Severe
Total (N)	14,783	517	185	216
Limb difficulties (In-Home Questionnaire / Parent Survey)	0 / 0	177 / 46	119 / 70	140 / 99
Use equipment for conditions (In-Home Questionnaire / Parent Survey)	0 / 0	259 / 0	97 / 18	114 / 51
Need aids for daily activities (In-Home Questionnaire / Parent Survey)	0 / 0	3 / 0	3 / 0	33 / 14
Self/Others consider disabled (In-Home Questionnaire / Parent Survey)	0 / 0	5 / 23	14 / 33	83 / 83
Difficulties in walking, standing, holding, grasping (In-Home Questionnaire)	0	20	46	101
More than 3 body parts affected (Parent Survey)	0	0	11	58
Blindness (one eye / both eyes)	0 / 0	0 / 0	0 / 0	16 / 3
Deafness	0	0	0	23

Among individuals with minimal disability (*n* = 517), the majority thought that they had limb difficulties (177, 34%) and used equipment for their conditions (259, 50%). On the other hand, very few people answered that they needed aids for daily activities (3 people), considered themselves disabled (5 people), and had difficulties walking, standing, holding, and grasping (20 people). There were no subjects with more than 3 body parts affected, blindness, or deafness. For people with mild disability, a higher proportion reported that they had any types of difficulties compared to individuals with minimal disability. Out of 185 people, 119 (64%), 97 (52%), and 46 (25%) responded that they had limb difficulties, used equipment for their conditions, and had difficulties walking, standing, holding, and grasping, respectively. Only a limited number of people considered themselves disabled (14 people). There were no people with blindness or deafness in this category. For individuals with moderate/severe disability (*n* = 216), 140 (65%), 114 (53%), 101 (47%), and 83 (38%) reported that they had limb difficulties, used equipment for their conditions, had difficulties walking, standing, holding, and grasping, and considered themselves disabled. Unlike the other categories, there were people with blindness (one eye: 16; both eyes: 3) and deafness (23 people) in this category.

The subjects’ demographic and socioeconomic information in Wave 4 is summarized in Table 2. This table includes age, race, sex, education level, and household income level. Age was consistent across the four disability/limitation statuses, with a mean age of 29 years (range: 28.5–28.9) and a SD of 1.7 years (range: 1.6–1.8). For race and sex, we found a slightly higher percentage of people with disabilities who were White (minimal: 3.6%; mild: 1.4%; moderate/severe: 1.6%) and male (minimal: 3.5%; mild: 1.3%; moderate/severe: 1.3%) compared with their counterparts, but the difference was not large. For education level, people with a high school diploma (minimal: 3.2%; mild: 1.1%; moderate/severe: 1.3%) were less likely to have a disability than those without a high school diploma (minimal: 4.0%; mild: 2.3%; moderate/severe: 2.2%). Similarly, those with high-income levels were less likely to have a disability than those with low- income levels. This trend was especially evident in the moderate/severe disability category (below/near poverty level: 2.6%; low-income: 1.6%; middle-income: 1.0%; high-income: 0.7%).

**Table 2:** Descriptive summary of demographic and socioeconomic variables by physical disability and/or limitation in Wave 4

Variables	Sub-variables	Total	No	Minimal	Mild	Moderate/ Severe
Total (N)	Population (count)	15,701	14,783	517	185	216
Age	Mean (year)	-	28.5	28.5	28.8	28.9
	Max (year)	-	34	33	33	33
	Min (year)	-	24	24	25	25
	SD (year)	-	1.8	1.7	1.6	1.6
Race	White (%)	10,945 (100)	10,231 (93.5)	393 (3.6)	149 (1.4)	172 (1.6)
	Black (%)	3,620 (100)	3,461(95.6)	95(2.6)	27 (0.7)	37 (1.0)
	American Indian or Alaska Native (%)	155 (100)	148 (95.5)	5 (3.2)	2 (1.3)	0 (0.0)
	Asian or Pacific Islander (%)	953 (100)	917 (96.2)	22 (2.3)	7 (0.7)	7 (0.7)
Sex	Male (%)	7,349 (100)	6,897 (93.8)	259 (3.5)	95 (1.3)	98 (1.3)
	Female (%)	8,352 (100)	7,886 (94.4)	258 (3.1)	90 (1.1)	118 (1.4)
Education	High school diploma (%)	14,675 (100)	13,845 (94.3)	475 (3.2)	161 (1.1)	194 (1.3)
	No high school diploma (%)	1,021 (100)	935 (91.6)	41 (4.0)	23 (2.3)	22 (2.2)
Household income	Below/near poverty level (less than $20 K) (%)	1,756 (100)	1,632 (92.9)	57 (3.2)	21 (1.2)	46 (2.6)
	Low-income ($20 K–50 K) (%)	4,801 (100)	4,491 (93.5)	167 (3.5)	67 (1.4)	76 (1.6)
	Middle-income ($5 K–150 K) (%)	7,349 (100)	6,965 (94.8)	237 (3.2)	72 (1.0)	75 (1.0)
	High-income (> $150 K) (%)	756 (100)	723 (95.6)	18 (2.4)	10 (1.3)	5 (0.7)

Table 3 shows the PA and PI levels of the adolescents (Wave 1) and young adults (Wave 4). In Wave 1, the mean PA level was approximately 3.5 times per week (range: 3.4–3.8) with a SD of 2.1 (range: 2.0–2.1) across the four levels of disability categories. On the other hand, the mean PI level was larger than the mean PA level, showing a mean of approximately 22.0 h per week (range: 20.9–23.0) with a SD of approximately 22.0 (range: 17.9–22.7). In Wave 4, no large difference was found for the mean PA level (range: 3.4–4.0) except for increases in the maximum values (range: 16.0–21.0). Even though the two survey datasets used different survey questionnaires for PI, we consistently observed decreases in the mean PI level (range: 15.4–19.3). For the differences between Wave 4 and Wave 1, we observed consistent decreases in PA sessions per week ranging from -4.1 to -6.8, while no large change was found from the mean PI hours per week (range: -0.2–0.4) regardless of disability level.

**Table 3:** Descriptive summary of the physical activity and physical inactivity levels in Waves 1 and 4 by physical disability and/or limitation

**Wave 1**		No	Minimal	Mild	Moderate/Severe
Physical activity (times per week)	Mean	3.6	3.8	3.6	3.4
	Max	9.0	9.0	8.0	9.0
	Min	0.0	0.0	0.0	0.0
	SD	2.1	2.1	2.1	2.0
Physical inactivity (hours per week)	Mean	23.0	22.5	20.9	23.5
	Max	282.0	200.0	159.0	110.0
	Min	0.0	0.0	0.0	0.0
	SD	21.6	22.7	22.1	17.9
**Wave 4**					
Physical activity (times per week)	Mean	3.6	3.6	4.0	3.4
	Max	21.0	17.0	16.0	17.0
	Min	0.0	0.0	0.0	0.0
	SD	3.1	3.0	3.5	3.1
Physical inactivity (hours per week)	Mean	16.2	17.5	15.4	19.3
	Max	198.0	160.0	124.0	126.0
	Min	0.0	0.0	0.0	0.0
	SD	16.4	18.3	15.9	20.3
**Differences between Wave 4 and Wave 1 (Wave 4—Wave 1)**					
Physical activity (times per week)	Mean	-6.8	-4.8	-5.3	-4.1
	Max	195	112	121	116
	Min	-268	-190	-152	-86
	SD	24.7	25.8	25.3	25.6
Physical inactivity (hours per week)	Mean	-0.0	-0.2	0.4	-0.0
	Max	18	13	12	15
	Min	-9	-9	-8	-6
	SD	3.4	3.4	3.6	3.1

### Associations between changes in PA/PI levels and demographic/socioeconomic variables

Here, we enumerate several statistically significant associations between changes in PA/PI levels and multiple demographic and socioeconomic variables and disability severity. Table 4 exhibits the RRR and p-values of each variable in the model.

**Table 4:** Multinomial logistic regression on changes in PA and PI levels by race, household income level, education level, sex, age, and physical disability and/or limitation. Relative risk ratios and *p*-values are shown in parentheses

Variables	Category/units	PA difference (Ref: No change)				PI difference (Ref: No change)			
		More decreased	Decreased	Increased	More increased	More decreased	Decreased	Increased	More increased
Race (Ref: White)	African American	0.83 (0.27)	1.07 (0.27)	0.97 (0.67)	1.05 (0.67)	4.08 (0.00)^c^	2.37 (0.00)^c^	1.35 (0.00)^c^	1.90 (0.00)^c^
	American Indian or Alaska Native	1.44 (0.54)	0.82 (0.50)	0.95 (0.86)	1.61 (0.18)	3.77 (0.00)^c^	1.72 (0.08)^a^	1.37 (0.35)	2.43 (0.06)^a^
	Asian or Pacific Islander	0.66 (0.22)	1.12 (0.31)	1.03 (0.77)	1.29 (0.12)	0.62 (0.15)	1.24 (0.13)	1.24 (0.13)	1.19 (0.53)
Household income (Ref: Below or near poverty level)	Low	0.80 (0.32)	1.09 (0.35)	1.10 (0.35)	0.82 (0.17)	0.77 (0.07)^a^	0.82 (0.05)^b^	0.74 (0.00)^c^	0.57 (0.00)^c^
	Middle	0.86 (0.50)	0.96 (0.68)	1.15 (0.16)	0.85 (0.23)	0.64 (0.00)^c^	0.66 (0.00)^c^	0.57 (0.00)^c^	0.35 (0.00)^c^
	High	1.69 (0.08)^a^	1.17 (0.25)	1.18 (0.27)	0.61 (0.05)^a^	0.37 (0.00)^c^	0.59 (0.00)^c^	0.50 (0.00)^c^	0.33 (0.00)^c^
Education (Ref: High school diploma)	No high school diploma	0.79 (0.36)	0.78 (0.01)^c^	1.14 (0.31)	1.07 (0.72)	0.76 (0.11)	0.91 (0.49)	0.86 (0.25)	0.58 (0.00)^c^
Sex (Ref: Male)	Female	0.38 (0.00)^c^	0.65 (0.00)^c^	0.82 (0.00)^c^	0.55 (0.00)^c^	0.48 (0.00)^c^	0.60 (0.00)^c^	0.65 (0.00)^c^	0.59 (0.00)^c^
Age	Continuous data	0.72 (0.00)^c^	0.84 (0.00)^c^	1.06 (0.00)^c^	1.11 (0.00)^c^	0.85 (0.00)^c^	0.89 (0.00)^c^	1.05 (0.02)^b^	0.98 (0.46)
Disability (Ref: No disability)	Minimal	0.96 (0.92)	1.29 (0.05)^b^	0.81 (0.23)	0.87 (0.59)	0.86 (0.61)	0.69 (0.08)^a^	0.96 (0.83)	1.31 (0.37)
	Mild	2.09 (0.11)	0.91 (0.71)	0.89 (0.66)	1.55 (0.17)	0.75 (0.58)	0.54 (0.12)	0.87 (0.67)	0.85 (0.78)
	Moderate/Severe	0.00 (0.00)^c^	0.80 (0.35)	0.69 (0.17)	0.86 (0.69)	0.89 (0.80)	1.54 (0.07)^a^	0.95 (0.86)	2.25 (0.02)^b^

### Race

Even though we were not able to observe any significant relationships between PA differences (Wave 4—Wave 1) and race at the 95% significance level, African American and American Indian or Alaskan Native individuals had a significant relationship with PI differences. In detail, African Americans were more likely to either increase (Increased: RRR = 1.35, *p*-value = 0.00; More increased: RRR = 1.90, *p*-value = 0.00) or decrease (Decreased: RRR = 2.37, *p*-value = 0.00; More decreased: RRR = 4.08, *p*-value = 0.00) their PI levels than maintain their PI levels compared to White individuals. On the other hand, American Indian or Alaskan Native individuals were more likely to decrease (more decreased: RRR = 3.77, *p*-value = 0.00) their PI levels than maintain their PI levels compared with White individuals.

### Household income

Household income was also one of the most important factors deciding PA and PI patterns over the life course. For the PA difference, although not statistically significant at the 95% significance level, we found that all income groups were more likely to increase (Low-income: RRR = 1.10, *p*-value = 0.35; Middle-income: RRR = 1.15, *p*-value = 0.16; High-income: RRR = 1.18, *p*-value = 0.27) their PA levels than maintain their PA levels compared with the group below or near the poverty level. However, we also found that the group below or near the poverty level was more likely to be classified in the “more increased” category than other income groups (Low-income: RRR = 0.82, *p*-value = 0.17; Middle-income: RRR = 0.85, *p*-value = 0.23; High-income: RRR = 0.61, *p*-value = 0.05). In other words, all income groups tended to increase their PA levels to a certain level and then decrease their PA levels compared to the group below or near the poverty level.

On the other hand, for the PI difference, we found that low-income groups (increased: RRR = 0.74, *p*-value = 0.00; more increased: RRR = 0.57, *p*-value = 0.00) were less likely to increase their PI levels than maintain their PI levels compared to the group below or near the poverty level. Interestingly, the middle- (more decreased: RRR = 0.64, *p*-value = 0.00; decreased: RRR = 0.66, *p*-value = 0.00; increased: RRR = 0.57, *p*-value = 0.00; more increased: RRR = 0.35, *p*-value = 0.00) and high-income groups (more decreased: RRR = 0.37, *p*-value = 0.00; decreased: RRR = 0.59, *p*-value = 0.00; increased: RRR = 0.50, *p*-value = 0.00; more increased: RRR = 0.33, *p*-value = 0.00) were more likely to maintain their PI levels than decrease or increase their PI levels compared to the group below or near the poverty level.

### Education

Individuals without high school diplomas were 0.78 times less likely to decrease their PA levels than maintain their PA levels compared to those with high school diplomas. Although not statistically significant, we found that people without high school diplomas were less likely to decrease their PA (more decreased: RRR = 0.79, *p*-value = 0.36; decreased: RRR = 0.78, *p*- value = 0.01) and more likely to increase their PA (increased: RRR = 1.14, *p*-value = 0.31; more increased: RRR = 1.07, *p*-value = 0.72) than maintain their PA levels compared to those with high school diplomas. On the other hand, for PI, we found that those without high school diplomas were less likely to increase their PI levels (more increased: RRR = 0.58, *p*-value = 0.00) than maintain their PI levels compared to those with high school diplomas.

### Sex and age

For sex, we found that females were more likely to maintain their PA levels (more decreased: RRR = 0.38, *p*-value = 0.00; decreased: RRR = 0.65, *p*-value = 0.00; Increased: RRR = 0.82, *p*-value = 0.00; more increased: RRR = 0.55, *p*-value = 0.00) and PI levels (more decreased: RRR = 0.48, *p*-value = 0.00; decreased: RRR = 0.60, *p*-value = 0.00; increased: RRR = 0.65, *p*-value = 0.00; more increased: RRR = 0.59, *p*-value = 0.00) than increase or decrease their PA levels compared to males. However, we found a different relationship with age. With increases in age, individuals were less likely to decrease their PA levels (more decreased: RRR = 0.72, *p*-value = 0.00; decreased: RRR = 0.84, *p*-value = 0.00) and more likely to increase their PA levels (increased: RRR = 1.06, *p*-value = 0.00; more increased: RRR = 1.11, *p*-value = 0.00) than to maintain their PA levels. Similarly, with increases in age, people were less likely to decrease their PI levels (more decreased: RRR = 0.85, *p*-value = 0.00; decreased: RRR = 0.89, *p*- value = 0.00) and more likely to increase their PI levels (increased: RRR = 1.05, *p*-value = 0.02) than to maintain their PA levels.

### Associations between changes in PA/PI levels and physical disability and/or limitation status

We found that individuals with minimal disabilities were more likely to decrease their PA levels (decreased: RRR = 1.29; *p*-value = 0.05) than to maintain their PA levels compared to those without disabilities (Table 4). This suggests that the group with the minimal level of disability was 1.29 times more likely to be in the decreased PA group than in the no difference PA group. For PI, we also found that individuals with moderate/severe disabilities were more likely to increase their PI levels (more increased: RRR = 2.25; *p*-value = 0.02) than maintain their PI levels compared to those without disabilities. This indicates that the group with a moderate/severe level of disability was 2.25 times more likely to belong to the more increased PI group than in the no difference PI group. However, no statistically significant impact was found for those with mild levels of disability (reference group: general population) in either PA or PI levels.

## Discussion

Our findings partially support that individuals with disabilities are more likely to reduce their PA engagement level than those without disabilities after their transition from adolescence to young adulthood. To the best of our knowledge, there are no or very limited studies examining PA level changes in people with disabilities between adolescence and adulthood. Additionally, no study has compared changes in PA levels between people with and without disabilities after the transition from adolescence to young adulthood. Our study is the first to provide important evidence indicating that people with disabilities may be more likely to reduce their PA levels than those without disabilities. There are at least two possible explanations for this finding. First, people with disabilities are more likely to face challenges when engaging in any type of PA during adulthood than during adolescence. Current education systems focus on providing students with disabilities more opportunities to interact with their friends [63]. Educators are thus encouraged to help students with disabilities engage in both academic and nonacademic activities to increase interactive experiences with their friends, which consequently brings greater acceptability and involvement of those with disabilities across all activities [64, 65]. However, adults with disabilities would have relatively lower acceptability and involvement after graduating from school. Second, individuals with disabilities are more likely to have physical- and mental health-related illnesses. With increasing age, people are more likely to experience functional limitations due to reduced muscular strength, imbalanced coordination, and joint-related problems, which are harmful for engaging in diverse daily activities [66, 67]. People with disabilities would experience higher chances of experiencing these problems due to their relatively limited physical conditions. Our finding suggests that we may need to allocate more resources to individuals with disabilities after the transition period.

We also found that individuals with moderate/severe disabilities were more likely to increase their PI levels when they transitioned from adolescence to young adulthood compared to those without disabilities. In general, people, regardless of their disability status, tend to have increased their PI time as they get older [68, 69]. However, individuals with moderate/severe disabilities are more likely to have more PI, such as watching television, videos and DVDs and playing computer and video games, than those without disabilities [70]. Our study is well aligned with this result, and our findings further showed that individuals with moderate/severe disabilities were more likely to increase their PI time when they were young adults compared to when they were adolescents. There are at least two explanations for why PI increases with age in individuals with disabilities. First, support for a variety of daily activities for adults with disabilities is relatively lacking compared to that for adolescents with disabilities [71]. Relatively limited support after graduation may prevent people with disabilities from performing PA and, consequently, encourage PI. Second, people's limited understanding of people with disabilities could discourage them from performing PA. People without disabilities often face difficulties when they interact with populations with disabilities. For example, the lack of knowledge of staff in PA settings leads to a disadvantage for older adults with disabilities, who require more appropriate exercise planning and implementation [72, 73]. Contrary to our results, a few studies indicated that there was no clear relationship between PI levels and the severity of disability [74, 75]. We suspect this difference may come from different types of disabilities, as both studies were based on people with intellectual and developmental disabilities.

Furthermore, we found that household income plays a significant role in deciding current PA levels. Our finding showed that individuals across all income groups (i.e., low, middle, high) were more likely to be classified into the “increased” PA level group and less likely to be classified into the “more increased” PA level group than the group below or near the poverty level. This suggests that people within low-, middle-, and high-income groups are more likely to increase their PA levels to a certain degree and then lower their PA levels compared to those in the group below or near the poverty level. This result can be partially supported by previous papers. Giles-Corti et al. (2015) and Kari et al. (2015) found that a high-income level is significantly related to a healthy lifestyle, such as having more PA participation and engagement in other health-related behaviors [37, 76]. Low-income is also regarded as a negative factor limiting daily activities, including PA, due to affordability and accessibility [77]. However, to the best of our knowledge, there have been no studies looking at the impact of income levels on the changes in PA levels from the perspective of the life course. Our study is the first to show evidence that people below or near the poverty level are more likely to spend considerable time over a certain level of PA than people in other income groups when they are young adults compared to when they are adolescents. We suspect this is related to time allocation. A previous study showed that students in financially disadvantaged minority groups reported extensive time spent hanging out with their friends [78]. This behavioral pattern may be maintained after they become young adults, but further research is needed to gain a better understanding of this relationship.

Our findings also indicate that people with high school diplomas are less likely to decrease their PA levels and increase their PI levels. This result is similar to previous studies that reported a positive relationship between PA engagement and education level [79]. We assume that major transitions during early adulthood (e.g., independent living) and related socioeconomic variables (e.g., income level, employment status) played an important role in changing an individuals’ PA pattern [51–53]. However, there is a lack of studies investigating individuals’ education and PI levels. We also found that as people age, they are less likely to decrease their PA and more likely to increase their PI levels. In general, PA levels decrease during the transition from adolescence to young adulthood [80, 81]. A recent review paper written by Hayes et al. (2019) also showed decreased PA and increased PI patterns (e.g., sedentary behavior) from adolescence to young adulthood [82]. There are many variables impacting health behaviors during this critical period that accompany many major changes in social relationships and academic and/or employment status. Future studies need to further examine what factors lead to decreased PA and increased PI levels.

Finally, we showed that females tend to maintain, rather than increase or decrease, their PA and PI levels between adolescence and young adulthood. This result is different from previous studies. Even though there is no consensus on whether females increase or decrease their PA and PI levels over the life course, most papers agree that females tend to change their PA and PI levels rather than maintain them during the transition. Armstrong et al. (2018) indicated that females across all racial and ethnic groups are less likely to maintain their PA levels from adolescence to young adulthood [17]. At the same time, Telama & Yang (2000) showed that boys tended to reduce their PA levels more than girls before 12 years of age [83]. They also reported that girls were more likely to be engaged in PA than boys after 18 years of age. We suspect this difference may come from different research population groups. Our study merely explored the PA and PI patterns targeting comparatively young age groups, excluding the older adult population. Follow-up studies using longer lifetime longitudinal data would further explain the relationship of PA and PI levels with age.

There are multiple future research opportunities. Our study did not compare the changes in PA and PI levels from adolescence to young adulthood between two same socioeconomic status groups (i.e., household income, education, gender) with and without disabilities. We only found that income and education are significantly associated with the changes in PA and PI levels regardless of disability status. Future studies could closely examine the differential impacts of various socioeconomic variables on the changes in PA and PI levels by disability status. For example, one might hypothesize that low-income people with disabilities could have lower PA engagement rates compared to low-income people without disabilities. Similarly, high-education people with disabilities could have higher PA engagement rates than low-education people without disabilities. More specific information on people with lower PA engagement rates would help prioritize resource allocations.

Future studies could also look at whether the type of disability is associated with the level of PA and PI. Previous studies have already found that people with visual impairments tend to have the least PA among people with disabilities [84]. Follow-up studies could separately investigate the level of PA and PI by different types of physical disabilities or limitations such as arm/hand impairment, leg/foot impairment, visual impairment, hearing impairment, and back impairment. In addition, researchers could further examine the relationship between physical disabilities and limitations and the types of PA and PI. In the present study, we only considered the total bouts or time used for all types of PA and PI. However, people with disabilities or limitations would have their own preferences towards different types of PA (e.g., individual sports: walking, roller-skating, bicycling; team sports: baseball, softball, basketball) or PI (e.g., watching television/videos; playing video or computer games).

Furthermore, Follow-up studies could look at other wave datasets to comprehensively understand PA and PI patterns depending on chronological age from adolescence to older adulthood. Next, our study only looked at the relationship between PA/PI levels and physical disability and/or limitation status. PA and PI levels are highly associated with a variety of socioeconomic, demographic, and psychological variables. Follow-up studies could look at other possible relationships between the variables (e.g., self-efficacy) and future PA or PI. Finally, we only quantitatively measured the number of hours spent on PA and PI to understand how disability influences future PA and PI levels. Although our results may suggest a simplified association between changes in PA/PI levels and disability status, our method does not provide a concrete explanation for why people with disabilities had decreased PA and increased PI levels. These questions can only be answered with qualitative studies or studies with mixed methods. More in-depth interviews or surveys are needed in future studies.

The strength of our results presented in this study lies in the use of large national longitudinal adolescent health data that track the same U.S. subjects at multiple points in time. However, there are several limitations in this study. First, our methods calculating PA and PI engagement levels could be less accurate, as Waves 1 and 4 used different types of questions to ask about PA and PI levels. Different questions between two surveys may introduce some errors or uncertainties in the analysis. Nevertheless, our method based on the relative values using SDs, rather than absolute values, may not have changed the relationships significantly. Second, we referred to previous studies to categorize the subjects into several categories based on disability severity. However, this classification method could be problematic, as it considers most types of disabilities as equal. Follow-up studies should apply proper weights to each type of disability to accurately explore the relationships between PA and PI levels accordingly. Third, a 1-week self-recall questionnaire to measure PA and PI levels could be subjective and biased. People’s memory capacity is different by age. This could lead to systematic differences between Wave 1 (adolescence) and Wave 4 (young adulthood) in terms of memory capacity. To overcome this flaw, future studies should use more objective tools, such as GPS, accelerometers, pedometers, and heart rate monitors, which would give more objective measurements on PA and PI levels rather than relying on people’s memories.

## Conclusion

We have at least four findings that need to be highlighted in this paper. First, this study partially supports that individuals with minimal disabilities were more likely to decrease their PA levels than those without disabilities during transitions from adolescence to young adulthood. Second, we showed that individuals with moderate to severe disabilities tended to have more PI hours than individuals without disabilities when they were young adults compared to when they were adolescents. Third, people in the low-, middle-, and high-income groups were more likely to increase their PA levels to a certain degree and then lower their PA levels compared to the group below or near the poverty level. Fourth, people with high school diplomas were less likely to decrease their PA levels and more likely to increase their PI levels than their counterparts.

There are at least two practical implications of the study results. We showed that people with disabilities tend to have decreased PA and increased PI engagement between transitions from adolescence to young adulthood. As noted by Block et al. (2007), individuals with physical disabilities and/or limitations often encounter multiple difficulties engaging in regular PA in that regular PA programs in the community generally do not provide fully accommodated programs for individuals with special needs [85]. Relatedly, federal or local governments should allocate more resources to professional development and training opportunities for preservice and in-service professionals who are working in diverse PA settings. Previous literature has already pointed out that staff in general PA settings in the community often lack the knowledge and training to work with populations with disabilities [86], which can result in failing to accommodate individuals with disabilities to have proper PA participation. Through practical training opportunities for these professionals, they can improve their knowledge and confidence in properly accommodating the special needs of individuals with disabilities in diverse PA environments. In the same context, training and/or disability awareness programs could be provided for the general population without disabilities so that they would not have any unfavorable attitudes toward populations with disabilities in PA settings. The literature often describes that there are some prejudices and unfavorable attitudes toward populations with disabilities in diverse PA settings [46, 85, 87].

Second, we revealed that people with disabilities who have low-income and low-education levels were more likely to have reduced PA than their counterparts. As noted by Moran & Martin (2010), there is often a lack of appropriate PA programs for populations with disabilities, which can originate from both financial reasons and a lack of human resources (e.g., a lack of experienced PA professionals working with individuals with special needs) [88]. Support systems for staff members in general PA settings and disability-related specialists, which would help them to more actively participate in diverse PA settings for people with disabilities, could possibly mitigate the health disparities between those with and without disabilities. Furthermore, public health agencies at the state and federal levels could provide free vouchers or transportation for those with low-SES to increase PA and decrease PI.

To ensure PA for all, PA professionals should be trained to have proper knowledge to provide appropriate PA programs to meet the unique needs of individuals with disabilities. Likewise, people without disabilities should better understand people with special needs by participating in trainings such as disability awareness training. At the same time, administrative and policy efforts should be made to solve the problem of access to sports facilities. By doing so, we can mitigate health disparities coming from systematic structures such as the individual and environmental constraints of minority groups.

## Supplementary Information

**Additional file 1: Table 1. **Questions used for collecting physicalactivity and physical inactivity in Wave 1 and Wave 4.

## Data Availability

The raw data and datasets used and/or analyzed for the current study are publicly available with security plan, IRB approval letter, and processing fee through https://addhealth.cpc.unc.edu/.

## Abbreviations

PA: Physical activity
PI: Physical inactivity
SES: Socioeconomic status

## References

[1] Miles L (2007). Physical activity and health. Nutr Bulletin.

[2] Reiner M, Niermann C, Jekauc D, Woll A (2013). Long-term health benefits of physical activity–a systematic review of longitudinal studies. BMC Public Health.

[3] Mota J, Fidalgo F, Silva R, Ribeiro JC, Santos R, Carvalho J (2008). Relationships between physical activity, obesity and meal frequency in adolescents. Ann Hum Biol.

[4] Duclos M, Oppert JM, Verges B, Coliche V, Gautier JF, Guezennec Y (2013). Physical activity and type 2 diabetes. Recommandations of the SFD (Francophone Diabetes Society) diabetes and physical activity working group. Diabetes Metab.

[5] Forrest KY, Bunker CH, Kriska AM, Ukoli FA, Huston SL, Markovic N (2001). Physical activity and cardiovascular risk factors in a developing population. Med Sci Sports Exerc.

[6] Carter MI, Hinton PS (2014). Physical activity and bone health. Mo Med.

[7] Larun L, Nordheim LV, Ekeland E, Hagen KB, Heian F. Exercise in prevention and treatment of anxiety and depression among children and young people. Cochrane Database Syst Rev. 2006 Jul 19;(3):CD004691. 10.1002/14651858.CD004691.pub2 PMC12742371 16856055

[8] Carlson SA, Adams EK, Yang Z, Fulton JE (2018). Percentage of deaths associated with inadequate physical activity in the United States. Prev Chronic Dis.

[9] Warburton DER, Bredin SSD (2016). Reflections on physical activity and health: What should we recommend?. Can J Cardiol.

[10] Hallal PC, Andersen LB, Bull FC, Guthold R, Haskell W, Ekelund U (2012). Global physical activity levels: surveillance progress, pitfalls, and prospects. Lancet.

[11] Bijlsma AY, Meskers MCG, Molendijk M, Westendorp RGJ, Sipilä S, Stenroth L (2013). Diagnostic measures for sarcopenia and bone mineral density. Osteoporos Int.

[12] Sillanpää E, Cheng S, Häkkinen K, Finni T, Walker S, Pesola A (2014). Body composition in 18- to 88-year-old adults–comparison of multifrequency bioimpedance and dual-energy X-ray absorptiometry. Obesity (Silver Spring).

[13] McPhee JS, French DP, Jackson D, Nazroo J, Pendleton N, Degens H (2016). Physical activity in older age: perspectives for healthy ageing and frailty. Biogerontology.

[14] Sherrington C, Fairhall N, Kwok W, Wallbank G, Tiedemann A, Michaleff ZA (2020). Evidence on physical activity and falls prevention for people aged 65+ years: systematic review to inform the WHO guidelines on physical activity and sedentary behaviour. Int J Behav Nutr Phys Act.

[15] Corder K, Winpenny E, Love R, Brown HE, White M, van Sluijs E (2019). Change in physical activity from adolescence to early adulthood: a systematic review and meta-analysis of longitudinal cohort studies. Br J Sports Med.

[16] Gordon-Larsen P, Nelson MC, Page P, Popkin BM (2006). Inequality in the built environment underlies key health disparities in physical activity and obesity. Pediatrics.

[17] Armstrong S, Wong CA, Perrin E, Page S, Sibley L, Skinner A (2018). Association of physical activity with income, race/ethnicity, and sex among adolescents and young adults in the United States: Findings from the National Health and Nutrition Examination Survey, 2007–2016. JAMA Pediatr.

[18] Azevedo MR, Araújo CLP, Reichert FF, Siqueira FV, da Silva MC, Hallal PC (2007). Gender differences in leisure-time physical activity. Int J Public Health.

[19] Biadgilign S, Gebremichael B, Abera A, Moges T (2022). Gender difference and correlates of physical activity among urban children and adolescents in Ethiopia: A cross-sectional study. Front Public Health.

[20] Telama R, Yang X, Leskinen E, Kankaanpää A, Hirvensalo M, Tammelin T (2014). Tracking of physical activity from early childhood through youth into adulthood. Med Sci Sports Exerc.

[21] Choi JY, Chang AK, Choi EJ (2015). Sex differences in social cognitive factors and physical activity in Korean college students. J Phys Ther Sci.

[22] Iannotti RJ, Chen R, Kololo H, Petronyte G, Haug E, Roberts C (2013). Motivations for adolescent participation in leisure-time physical activity: international differences. J Phys Act Health.

[23] Troiano RP, Berrigan D, Dodd KW, Mâsse LC, Tilert T, McDowell M (2008). Physical activity in the United States measured by accelerometer. Med Sci Sports Exerc.

[24] Sallis JF, Zakarian JM, Hovell MF, Hofstetter CR (1996). Ethnic, socioeconomic, and sex differences in physical activity among adolescents. J Clin Epidemiol.

[25] Belcher BR, Berrigan D, Dodd KW, Emken BA, Chou CP, Spruijt-Metz D (2010). Physical activity in US youth: effect of race/ethnicity, age, gender, and weight status. Med Sci Sports Exerc.

[26] Li R, Sit CHP, Yu JJ, Duan JZJ, Fan TCM, McKenzie TL (2016). Correlates of physical activity in children and adolescents with physical disabilities: A systematic review. Prev Med.

[27] Kao D, Carvalho Gulati A, Lee RE (2016). Physical activity among Asian American adults in Houston, Texas: Data from the Health of Houston Survey 2010. J Immigr Minor Health.

[28] Towne SD, Ory MG, Smith ML, Peres SC, Pickens AW, Mehta RK (2017). Accessing physical activity among young adults attending a university: the role of sex, race/ethnicity, technology use, and sleep. BMC Public Health.

[29] Singh GK, Yu SM, Siahpush M, Kogan MD (2008). High levels of physical inactivity and sedentary behaviors among US immigrant children and adolescents. Arch Pediatr Adolesc Med.

[30] Go AS, Mozaffarian D, Roger VL, Benjamin EJ, Berry JD, Blaha MJ (2014). Heart disease and stroke statistics–2014 update: a report from the American Heart Association. Circulation.

[31] Sebastião E, Ibe-Lamberts K, Bobitt J, Schwingel A, Chodzko-Zajko W (2014). Employing a participatory research approach to explore physical activity among older African American women. J Aging Res.

[32] Bantham A, Taverno Ross SE, Sebastião E, Hall G (2021). Overcoming barriers to physical activity in underserved populations. Prog Cardiovasc Dis.

[33] Kim J. The role of social ecological factors in shaping leisure time physical activity and mental health among Asian immigrants in the United States [dissertation]. [Pennsylvania]: The Pennsylvania State University; 2018.

[34] McArthur LH, Raedeke TD (2009). Race and sex differences in college student physical activity correlates. Am J Health Behav.

[35] Walcott-McQuigg JA, Sullivan J, Dan A, Logan B (1995). Psychosocial factors influencing weight control behavior of African American women. West J Nurs Res.

[36] Giles-Corti B, Donovan RJ (2002). Socioeconomic status differences in recreational physical activity levels and real and perceived access to a supportive physical environment. Prev Med.

[37] Kari JT, Pehkonen J, Hirvensalo M, Yang X, Hutri-Kähönen N, Raitakari OT, et al. Income and physical activity among adults: Evidence from self-reported and pedometer-based physical activity measurements. Harezlak J, editor. PLoS ONE. 2015 Aug 28;10(8):e0135651. 10.1371/journal.pone.0135651 PMC4552741 26317865

[38] Mendoza-Vasconez AS, Linke S, Muñoz M, Pekmezi D, Ainsworth C, Cano M (2016). Promoting physical activity among underserved populations. Curr Sports Med Rep.

[39] Li L, Moosbrugger ME (2021). Correlations between physical activity participation and the environment in children and adolescents: A systematic review and meta-analysis using ecological frameworks. Int J Environ Res Public Health.

[40] Li M, Dibley MJ, Sibbritt D, Yan H (2006). Factors associated with adolescents’ physical inactivity in Xi’an City. China Med Sci Sports Exerc.

[41] Muñoz-Galiano IM, Connor JD, Gómez-Ruano MA, Torres-Luque G (2020). Influence of the parental educational level on physical activity in schoolchildren. Sustainability.

[42] Liou TH, Pi-Sunyer FX, Laferrère B (2005). Physical disability and obesity. Nutr Rev.

[43] Buffart LM, Westendorp T, van den Berg-Emons RJ, Stam HJ, Roebroeck ME (2009). Perceived barriers to and facilitators of physical activity in young adults with childhood-onset physical disabilities. J Rehabil Med.

[44] de Hollander EL, Proper KI (2018). Physical activity levels of adults with various physical disabilities. Prev Med Rep.

[45] Marmeleira J, Laranjo L, Marques O, Pereira C (2014). Physical activity patterns in adults who are blind as assessed by accelerometry. Adapt Phys Activ Q.

[46] Rimmer JH, Riley B, Wang E, Rauworth A, Jurkowski J (2004). Physical activity participation among persons with disabilities: barriers and facilitators. Am J Prev Med.

[47] McAuley E, Blissmer B (2000). Self-efficacy determinants and consequences of physical activity. Exerc Sport Sci Rev.

[48] Bloemen MAT, Backx FJG, Takken T, Wittink H, Benner J, Mollema J (2015). Factors associated with physical activity in children and adolescents with a physical disability: a systematic review. Dev Med Child Neurol.

[49] Clarke P, Latham K (2014). Life course health and socioeconomic profiles of Americans aging with disability. Disabil Health J.

[50] Prizer LP, Gay JL, Gerst-Emerson K, Froehlich-Grobe K (2016). The role of age in moderating the association between disability and light-intensity physical activity. Am J Health Promot.

[51] Hull EE, Rofey DL, Robertson RJ, Nagle EF, Otto AD, Aaron DJ (2010). Influence of marriage and parenthood on physical activity: a 2-year prospective analysis. J Phys Act Health.

[52] Rapp I, Schneider B (2013). The impacts of marriage, cohabitation and dating relationships on weekly self-reported physical activity in Germany: a 19-year longitudinal study. Soc Sci Med.

[53] Stenholm S, Pulakka A, Kawachi I, Oksanen T, Halonen JI, Aalto V (2016). Changes in physical activity during transition to retirement: a cohort study. Int J Behav Nutr Phys Act.

[54] Tabachnick BG, Fidell LS, Ullman JB. Using multivariate statistics. Boston, MA: pearson; 2007 Mar 3.

[55] Bayaga A (2010). Multinomial Logistic Regression: Usage and application in risk analysis. J Appl Quant Methods.

[56] Cheng MM, Udry JR (2002). Sexual behaviors of physically disabled adolescents in the United States. J Adolesc Health.

[57] McRee  AL, Haydon AA, Halpern  CT (2010). Reproductive health of young adults with physical disabilities in the U.. Prev Med.

[58] Gordon-Larsen P, Adair LS, Popkin BM (2002). Ethnic differences in physical activity and inactivity patterns and overweight status. Obes Res.

[59] Gordon-Larsen P, McMurray RG, Popkin BM (2000). Determinants of adolescent physical activity and inactivity patterns. Pediatr.

[60] Gordon-Larsen P, McMurray RG, Popkin BM (1999). Adolescent physical activity and inactivity vary by ethnicity: The National Longitudinal Study of Adolescent Health. J Pediatr.

[61] Ornelas IJ, Perreira KM, Ayala GX (2007). Parental influences on adolescent physical activity: A longitudinal study. Int J Behav Nutr Phys Act.

[62] Thivel D, Tremblay A, Genin PM, Panahi S, Rivière D, Duclos M. Physical activity, inactivity, and sedentary behaviors: definitions and implications in occupational health. Frontiers in Public Health. 2018 Oct;5;(6):288. 10.3389/fpubh.2018.00288 PMC6182813 30345266

[63] Maxey M, Beckert TE (2017). Adolescents with disabilities. Adolescent Res Rev.

[64] Ryndak DL, Jackson L, Billingsley F (2000). Defining school inclusion for students with moderate to severe disabilities: What do experts say?. Exceptionality.

[65] Murphy NA, Carbone PS (2008). American academy of pediatrics council on children with disabilities. promoting the participation of children with disabilities in sports, recreation, and physical activities. Pediatrics.

[66] Paterson DH, Warburton DE (2010). Physical activity and functional limitations in older adults: a systematic review related to Canada’s Physical Activity Guidelines. Int J Behav Nutr Phys Act.

[67] Spirduso WW, Cronin DL. Exercise dose-response effects on quality of life and independent living in older adults. Med Sci Sports Exerc. 2001 Jun;33(6 Suppl):S598–608; discussion S609–610. 10.1097/00005768-200106001-00028 11427784

[68] Kruger J, Kohl HW, Miles IJ (2007). Prevalence of regular physical activity among adults-United States, 2001 and 2005. Morb Mortal Wkly Rep.

[69] Motl RW, McAuley E (2010). Physical activity, disability, and quality of life in older adults. Phys Med Rehabil Clin N Am.

[70] Law M, Anaby D, Teplicky R, Khetani MA, Coster W, Bedell G (2013). Participation in the home environment among children and youth with and without disabilities. Br J Occup Ther.

[71] van Schijndel-Speet M, Evenhuis HM, van Wijck R, van Empelen P, Echteld MA (2014). Facilitators and barriers to physical activity as perceived by older adults with intellectual disability. Intellect Dev Disabil.

[72] Evenhuis HM, Hermans H, Hilgenkamp TIM, Bastiaanse LP, Echteld MA (2012). Frailty and disability in older adults with intellectual disabilities: results from the healthy ageing and intellectual disability study. J Am Geriatr Soc.

[73] Temple VA, Frey GC, Stanish HI (2006). Physical activity of adults with mental retardation: review and research needs. Am J Health Promot.

[74] Hsieh K, Hilgenkamp TIM, Murthy S, Heller T, Rimmer JH (2017). Low levels of physical activity and sedentary behavior in adults with intellectual disabilities. Int J Environ Res Public Health.

[75] Oppewal A, Hilgenkamp TIM, Schäfer Elinder L, Freiberger E, Rintala P, Guerra-Balic M (2018). Correlates of Sedentary Behaviour in adults with intellectual disabilities-A systematic review. Int J Environ Res Public Health.

[76] Giles-Corti B, Sallis JF, Sugiyama T, Frank LD, Lowe M, Owen N (2015). Translating active living research into policy and practice: one important pathway to chronic disease prevention. J Public Health Policy.

[77] Finkelstein DM, Petersen DM, Schottenfeld LS (2017). Promoting children’s physical activity in low-income communities in Colorado: What are the barriers and opportunities?. Prev Chronic Dis.

[78] Shann MH (2001). Students' use of time outside of school: A case for after school programs for urban middle school youth. Urban Rev.

[79] Scholes S, Bann D (2018). Education-related disparities in reported physical activity during leisure-time, active transportation, and work among US adults: repeated cross-sectional analysis from the National Health and Nutrition Examination Surveys, 2007 to 2016. BMC Public Health.

[80] Aira T, Vasankari T, Heinonen OJ, Korpelainen R, Kotkajuuri J, Parkkari J (2021). Physical activity from adolescence to young adulthood: patterns of change, and their associations with activity domains and sedentary time. Int J Behav Nutr Phys Act.

[81] Kjønniksen L, Torsheim T, Wold B (2008). Tracking of leisure-time physical activity during adolescence and young adulthood: a 10-year longitudinal study. Int J Behav Nutr Phys Act.

[82] Hayes G, Dowd KP, MacDonncha C, Donnelly AE (2019). Tracking of physical activity and sedentary behavior from adolescence to young adulthood: A systematic literature review. J Adolesc Health.

[83] Telama R, Yang X (2000). Decline of physical activity from youth to young adulthood in Finland. Med Sci Sports Exerc.

[84] Longmuir PE, Bar-Or O (2000). Factors influencing the physical activity levels of youths with physical and sensory disabilities. Adapt Phys Activ Q.

[85] Block ME, Obrusnikova I (2007). Inclusion in physical education: a review of the literature from 1995–2005. Adapt Phys Activ Q.

[86] Block ME. A teacher's guide to adapted physical education. Paul H. Brookes Publishing.; 2016.

[87] Martin Ginis KA, Ma JK, Latimer-Cheung AE, Rimmer JH (2016). A systematic review of review articles addressing factors related to physical activity participation among children and adults with physical disabilities. Health Psychol Rev.

[88] Moran T, Martin B (2010). Barriers to participation of children with disabilities in youth sports. Teaching Exceptional Children Plus.

