# Supplementary material for: How disability severity is associated with changes in physical activity and inactivity from adolescence to young adulthood
Source: Arch Public Health. 2023 Feb 21;81:29. doi: 10.1186/s13690-023-01043-0 (PMC9942288; doi:10.1186/s13690-023-01043-0)
Supplement: Supplementary file 1 — Additional file 1: Table 1. Questions used for collecting physicalactivity and physical inactivity in Wave 1 and Wave 4. [file 13690_2023_1043_MOESM1_ESM.docx]

| Category | Questionnaire type | Code | Question |
| --- | --- | --- | --- |
| Physical activity | Wave 1 | H1DA4 | During the past week, how many times did you go roller-blading, roller-skating, skate-boarding, or bicycling? |
|  |  | H1DA5 | During the past week, how many times did you play an active sport, such as baseball, softball, basketball, soccer, swimming, or football? |
|  |  | H1DA6 | During the past week, how many times did you do exercise, such as jogging, walking, karate, jumping rope, gymnastics or dancing? |
|  | Wave 4 | H4DA2 | In the past seven days, how many times did you bicycle, skateboard, dance, hike, hunt, or do yard work? |
|  |  | H4DA3 | In the past seven days, how many times did you roller blade, roller skate, downhill ski, snow board, play racquet sports, or do aerobics? |
|  |  | H4DA4 | In the past seven days, how many times did you participate in strenuous team sports such as football, soccer, basketball, lacrosse, rugby, field hockey, or ice hockey? |
|  |  | H4DA5 | In the past seven days, how many times did you participate in individual sports such as running, wrestling, swimming, cross-country skiing, cycle racing, or martial arts? |
|  |  | H4DA6 | In the past seven days, how many times did you participate in gymnastics, weight lifting, or strength training? |
|  |  | H4DA7 | In the past seven days, how many times did you play golf, go fishing or bowling, or play softball or baseball? |
|  |  | H4DA8 | In the past seven days, how many times did you walk for exercise? |
| Physical inactivity | Wave 1 | H1DA8 | How many hours a week do you watch television? |
|  |  | H1DA9 | How many hours a week do you watch videos? |
|  |  | H1DA10 | How many hours a week do you play video or computer games? |
|  |  | H1DA11 | How many hours a week do you listen to the radio? |
|  | Wave 4 | H4DA1 | In the past seven days, how many hours did you watch television or videos, including VHS, DVDs or music videos? |

Supplementary Table 1. Questions used for collecting physical activity and physical inactivity in Wave 1 and Wave 4
